# Can differences in individual learning explain patterns of technology adoption? Evidence on heterogeneous learning patterns and hybrid rice adoption in Bihar, India

**DOI:** 10.1016/j.worlddev.2018.11.014

**Published:** 2019-03

**Authors:** Jared Gars, Patrick S. Ward

**Affiliations:** a Organization for Economic Cooperation and Development (OECD), France; b Duke Kunshan University, China

**Keywords:** Learning heuristics, Experimental economics, Technology adoption, India

## Abstract

Much empirical research that has shown that an individual’s decision to adopt a new technology is the result of learning – both in personal experimentation as well as observing the experimentation of others. Yet even casual observation would suggest significant heterogeneity learning processes, manifesting itself in widely varying patterns of adoption over space and time. In this paper we explore this heterogeneity in the context of early adoption of hybrid rice in rural India. Using specially-designed experiments conducted as part of a primary survey in the field, we are able to identify which of four broad learning heuristics most accurately reflects individuals’ information processing strategies. Linking these learning heuristics with observed use of rice hybrids, we demonstrate that pure Bayesian learning is well suited for the tinkering and marginal adjustments that would be required to learn about a technology like hybrid rice, but is also more cognitively taxing, requiring a longer memory and more complex updating processes. Consequently, only about 25 percent of the farmers in our sample can be characterized as pure Bayesian learners. Present-biased learning and relying on first impressions will likely hinder adoption of a technology like hybrid rice, even after controlling for access to credit and a rudimentary proxy for intelligence.

## Highlights

• We use field experiments in India to understand how farmers process information.
• Most farmers simplify judgments by using only a portion of the information set.
• Nearly 40% of farmers rely on first impressions to make subsequent judgments.
• More than 36% of farmers use only the most recent piece of information.
• Farmers who simplify judgments are less likely to cultivate rice hybrids.

## 1 Introduction

In many parts of the developing world, the transition from indigenous agricultural practices to modern technologies is often viewed as a critical step toward achieving broad agricultural development objectives such as food security or self-sufficiency. Yet the process of technology adoption is often inconsistent over space and time. Clearly, differences in resource constraints including access to credit, labor availability, and land ownership affect individual adoption decisions. Furthermore, various studies have also attributed some of the heterogeneity in technology adoption to idiosyncratic differences in farmers’ risk preferences (e.g. Feder, 1980, Feder et al., 1985). In the context of modern seed varieties during the Green Revolution, Foster and Rosenzweig (1995) have demonstrated the importance of learning, both from one’s own experimentation as well as in observing the experimentation of others. This process involves an iterative process of forming and updating beliefs about yield or profit distributions, with new information gleaned from one’s own experimentation or form observing the experimentation of others contributing to the refined beliefs.

There is a considerable literature that has addressed how individuals process information and update beliefs in repeated decisionmaking, although much of this previous literature has been confined to the lab. Previous research on belief updating heuristics has found that subjects in the lab, including farmers in the US, exhibit substantial heterogeneity in how they update beliefs and that this heterogeneity is partially explained by observable levels of education and cognitive ability (Barham et al., 2015, Cheung and Friedman, 1997, Camerer and Ho, 1999, Gans et al., 2007).

But with the exception of Barham et al. (2015), however, there has been little work analyzing how individual learning heuristics vary across actual decision makers and how these heterogeneous learning heuristics differentially affect technology adoption decisions. Barham et al. (2015) find that farmers in Minnesota and Wisconsin tend to simplify information processing, and that farmers that form strong beliefs are slower adopters of new technologies, namely genetically modified (GM) corn and soybeans. The present study contributes to this literature by expanding the empirical approach of Barham et al. (2015) to agricultural settings in a developing country – namely India – and uncovering some of the broad heuristics through which Indian farmers form and update expectations about conditional probability distributions that facilitate or constrain the process of technology adoption – specifically hybrid rice. We utilize field experiments to elicit and characterize individual learning styles, and combine these findings with risk and uncertainty preferences and observed adoption decisions to test whether heterogeneity in learning acts as a significant barrier to adoption.

To foreshadow our results, we find that pure Bayesian learning is well suited for the tinkering and marginal adjustments that would be required to learn about a technology like hybrid rice. Nevertheless, Bayesian learning is also more cognitively taxing, requiring a longer memory and more complex updating processes. Consequently, only about 25 percent of the farmers in our sample can be characterized as pure Bayesian learners, compared with the nearly 40 percent of our sample who can most accurately be characterized as relying upon first impressions. Present-biased learning and relying on first impressions will likely hinder adoption of a technology like hybrid rice, even after controlling for access to credit and a rudimentary proxy for intelligence.

The remainder of the paper is organized as follows. In Section 2, we provide some contextual background, particularly on the history of varietal modernization during the Green Revolution in India, as well as a review of the behavioral economics and psychological literature on information processing and learning. In Section 3, we introduce our empirical strategy for addressing the question of learning heterogeneity and its implications for technology adoption, including a description of our experimental design for eliciting learning heuristics and a description of the four broad learning heuristics under examination. In Section 4, we present our results, including identification and ranking of most likely learning rules and their impacts on technology adoption. Finally, in Section 5, we offer some concluding remarks and suggest policy implications of our findings.

## 2 Contextual background

### 2.1 Background on varietal modernization during the Green Revolution

Experiences from the Asian Green Revolution from the mid-1960s through the end of the 1990s provide an almost textbook illustration of the transition from indigenous practices to modern technologies and the subsequent attainment of improved food security (e.g. Gollin, Morris, & Byerlee, 2005). Initially, during the early years of the Green Revolution, most of the revolution was with respect to the transition from traditional varieties and landraces to modern, high yielding varieties, specifically the adoption of semidwarf varieties of rice and wheat. From 1965 to 1970, estimates suggest that the cultivation of modern varieties of rice and wheat in South Asia had increased from an essentially negligible baseline to 10 percent and 39 percent of harvested area under, respectively (Gollin et al., 2005). In India, as in much of South Asia, the adoption of these modern varieties provided the potential for increased yields, though arguably these new varieties did not reach their full yield potential until they were paired with complementary inputs, such as irrigation and fertilizers. Indeed, the diffusion of these modern varieties in many ways propelled the adoption of fertilizers and irrigation (Gollin et al., 2005, Morris and Byerlee, 1998). But while the expansion of irrigation facilities, the increased use of chemical fertilizers and pesticides share some of the credit for the massive gains in foodgrain production during this period, such gains arguably might never have been possible without the widespread adoption of high-yielding rice and wheat varieties that were particularly responsive to these other complementary inputs (e.g. Morris & Byerlee, 1998).

This process of varietal modernization, however, has been inconsistent over both space and time. Most of the benefits of these modern varieties were realized in the northwestern states of Punjab, Haryana, and western Uttar Pradesh, who had larger and more egalitarian farm structures and who generally had greater access to irrigation, in some cases due to a more favorable policy environment. In other parts of India – particularly eastern Indian states such as Bihar, Odisha, and West Bengal – varietal modernization has been considerably slower. Furthermore, while there was initially quite rapid adoption of these modern varieties, the cumulative level of adoption, especially for rice, remains incomplete to this day. And even where farmers have made the transition from traditional varieties or landraces to modern varieties (what Morris & Byerlee, 1998 refer to as *Type A* varietal change), there has not necessarily been a subsequent transition from first generation modern varieties to newer modern varieties (what Morris & Byerlee, 1998 refer to as *Type B* varietal change). This latter form of varietal modernization has been shown to be particularly important, as many of the genetic advantages conferred by the breeding efforts deteriorate over time, resulting in reduced productivity and increased susceptibility to various stresses (Krishna, Spielman, & Veettil, 2016). Furthermore, more rapid varietal turnover among modern varieties allows farmers to access to technological enhancements embodied in newer germplasm. As the private seed sector continues to develop within India, this varietal turnover will likely take the form of switching from inbred varieties to hybrids, which offer higher yields and lower seed rates, though at the expense of farmers’ being able to save seed from year to year.

It has often been observed that the adoption of new technologies, including new cultivars, is a gradual process. When plotted against time, the cumulative proportion of a population adopting a given technology generally follows a sigmoid (S-shaped) trend, where the most rapid adoption (i.e., at an exponential growth rate) occurs at some non-trivial time after the initial introduction of a technology (Griliches, 1957, Rogers, 1995). The shape of the diffusion curve implies that a great deal of heterogeneity, which manifests itself through differentials in the timing of adoption among farmers in the population (e.g., some farmers are innovators, some are laggards, etc.). If one considers differences in the shape and the location of such diffusion curves across subgroups in the population (e.g., across different states), there emerges an even greater degree of cross-sectional heterogeneity, not just in terms of differences in the timing of adoption (i.e., what Griliches, 1957 refers to as the ‘date of origin’) in these different subgroups, but also the pace of adoption (i.e., the ‘rate of acceptance’) and the timing and cumulative level of steady state or equilibrium adoption rates.

Clearly, differences in resource constraints including access to credit, labor availability, and land ownership affect individual adoption decisions. Additionally, various studies also attribute this heterogeneity to idiosyncratic differences in farmers’ risk preferences (e.g. Feder, 1980, Feder et al., 1985). When considering a new technology, farmers are confronted with considerable uncertainty, since not only is there risk associated with the yield or profitability of the technology, but also because the nature of this risk (i.e., the underlying distribution of yields or potential farm profits) is unknown. The decision to transition from a traditional technology to a new technology requires some consideration of the relative benefits of the two.^1^ Under this view, therefore, there must be some process or mechanism by which expectations of the long-term value of the technology is uncovered or inferred. This, in turn, implies that a precondition for technology adoption is that the potential adopter has, in some fashion or another, learned about the potential benefits of the new technology, compared these benefits with those of the traditional technology, and considered the cost of transitioning from the traditional to the modern technology.

In the context of modern seed varieties during the Green Revolution, Foster and Rosenzweig (1995) have demonstrated the importance of learning, both from one’s own experimentation as well as in observing the experimentation of others. They suggest that imperfect or suboptimal input usage was the result of imperfect subjective beliefs, which were subsequently improved through Bayesian updating with increased experience with the new varieties. The profitability of modern varieties was increasing in own and neighbor experience with the modern varieties, but farmers with relatively richer neighbors were more likely to delay adoption and observe the costly experimentation of their neighbors. Foster and Rosenzweig (1995) suggest that these neighbor effects result in free-riding on the experiences of more capitalized farmers with respect to modern seed varieties in India. In their study of Mozambican farmers, Bandiera and Rasul (2006) find an inverse U-shaped propensity to adopt relative to the numbers of family and friends who adopt. There is an increasing network effect when there are relatively few adopters in the network, but as the number of adopters increases, the propensity to adopt declines, an effect attributed to strategic delay. In both cases, there is apparently a conflict between the uncovering of additional information on the benefits of the new technology and the temptation to delay one’s own adoption until the full distribution of potential benefits is realized: more information is helpful in proving optimal input information, but having multiple observations potentially allows farmers to wait and observe heterogeneous outcomes before experimenting on their own.

Heterogeneity in individual learning may influence how farmers weigh information from others and encourage or inhibit social learning. Conley and Udry (2010) consider the issue of information quality in social learning, and allow for a more flexible learning model that does not force farmers to learn an entire production function. Rather, they engage in local learning, or just learning about the relevant outcomes of the production function for the level of inputs that are applied. Munshi (2004) exploits heterogeneity in growing conditions amongst rice farmers during the Green Revolution to show that rice farmers are more likely to experiment than wheat farmers, as the quality of social information is considerably lower. In contrast, wheat farmers responded strongly to neighbors’ experiences as well as targeted extension efforts with groups of contact farmers. Social learning has the capacity to overcome challenges faced by farmers in the adoption decisions, though there remain groups of farmers that rely on learning by doing and individual experimentation on both the intensive and extensive margin when making adoption decision and intensity choices. Additionally, longer and more heterogeneous processes of diversification and adaptation to climate change may limit the scope for farmers to learn from their neighbors, forcing many farmers to rely on individual learning and experimentation.

### 2.2 Background on information processing and learning

Farmer adoption decisions rely on farmers processing information about the productivity or profitability of various technologies, updating their beliefs given this new information, and subsequently making choices based on their posterior, subjective distributions. Identifying sources of heterogeneity in intensity and timing of the adoption of productive technologies has been the focus of empirical research for decades (Feder, 1980, Foster and Rosenzweig, 1995, Griliches, 1957). The technology adoption literature has typically assumed Bayesian learning as it is empirically tractable and theoretically consistent (Conley and Udry, 2010, Foster and Rosenzweig, 1995). However, this is clearly an unrealistic assumption in almost any real-world context. Voluminous research suggests that individuals simplify otherwise complex cognitive tasks (e.g. Tversky & Kahneman, 1974), and furthermore that there is substantial heterogeneity in learning rules or heuristics that people employ which may affect the adoption decision and interact with other characteristics (i.e. risk preferences) to encourage or prevent adoption (Barham et al., 2015, Gans et al., 2007). The literature on learning in non-cooperative games has adapted a variety of models of learning and updating beliefs which may be more relevant in contexts where there are low levels of human capital (Cheung and Friedman, 1997, Camerer and Ho, 1999).

Differences in non-Bayesian updating or learning are the result of how people process or weight the information at their disposal. Belief learning models characterize how players update beliefs and make decisions given their subjective expected distribution based on observed history of outcomes. Two common belief learning models are Cournot learning (short memory) and fictitious play (long memory). Cheung and Friedman (1997) develop a general one parameter class of learning rules (weighted fictitious play) that nests Cournot and fictitious play as special cases, with a range between them of adaptive learning rules whereby all observations may affect the expected state but the weight given to more recent information varies with the parameter. Importantly, Cheung and Friedman (1997) find that players exhibit a range of learning styles and that players are more likely to weight recent information in more informative environments. Camerer and Ho (1999) introduced a hybrid model of learning that includes aspects of both belief learning and reinforcement or rote learning. The Experience Weighted Attraction (EWA) model “wraps a parametric skin” around belief and reinforcement learning as boundaries of the parameter space. However, their model may have little relevance in purely individual games where payoffs from other strategies are stochastic and not well known.

The learning models described above belong to the set of individual learning models, or what may be considered behavioral models of “learning by doing.” Other prominent individual learning models include reinforcement learning (RL) and individual evolutionary learning (IEL) which have applications in a variety of settings but may be less relevant for technology adoption (Arifovic and Ledyard, 2011, Erev and Roth, 1998). Our study seeks to identify heterogeneous learning styles and determine their effect on technology adoption decisions where farmers make choices over multiple technologies which have unknown yield or profit distributions. The farmer’s problem then is similar to two-armed or multi-armed bandit problems in which farmers are optimizing their decisions while simultaneously improving their information. The farmers face the inherent trade-off between experimentation (i.e., on a temporary and reversible basis) and adoption (i.e., on a more permanent and irreversible basis). Results from multi-armed bandit experiments provide evidence that people diverge from Bayesian behavior. Meyer and Shi (1995) find that players tend to be myopic in their updating and make less than optimal decisions due to this updating. Anderson (2001) attributes less than optimal experimentation to risk aversion. Gans et al. (2007) evaluate multiple simple updating rules relative to the Gittins Index and find substantial heterogeneity in peoples’ updating rules and that they outperform the optimal decisions given the environment.

Barham et al. (2015) develop a learning experiment where subjects choose between a sure and risky payoff as they obtain more information about the underlying probability distribution of “winning” and “losing” draws in a risky lottery, after a series of draws with replacement. They evaluate the learning rules used by Gans et al. (2007) and find evidence that farmers in their sample tend to be most influenced by recent draws but conclude there is heterogeneity in learning rules, as some farmers are closer to the type of behavioral that would be predicted by strict Bayesian updating. Farmers with higher levels of education were more likely to use more sophisticated learning rules, suggesting that human capital may diminish the impacts of suboptimal learning. Integrating their experimental results with Wisconsin farmers’ recollection of the timing of their adoption genetically modified maize and soybeans, they find that farmers that develop strong beliefs (such as being sensitive to first impressions) are slower adopters, as they neglect continual learning.

## 3 Empirical strategy

In an effort to shed light on the impact of learning on technology adoption in rural Bihar, our empirical tests take advantage of a multi-year panel data set of 576 farmers in rural India. From the first data round in 2013, farmers’ risk and uncertainty preferences were elicited using lottery based experiments and used to calibrate a learning experiment in the second round in 2014. In the second round, farmers participated in a learning experiment where they again made a series of decisions between a lottery and a riskless payout. The payoffs from the lottery and the riskless payout remained constant throughout the experiment but each round revealed more information about the underlying distribution of risk in the lottery as the farmer drew five beads (with replacement) from a bag in each round. Their actual payoffs in the lottery were based on the true distribution of beads but their per round expected utility payoffs were based on their updated beliefs as more information was revealed. We collected plot level inputs and yields in both survey rounds, as well as individual characteristics including age, caste, and literacy that potentially affect the learning and technology adoption process. Preliminary results from the learning experiments reveal considerable heterogeneity in learning across farmers, assuming both risk neutrality and risk aversion. In the case of risk neutrality, we find that Bayesian learning is not an inappropriate model of belief updating for many farmers, but over-weighting early events is a better approximation of learning regardless of whether risk neutrality or risk aversion are assumed. We also find evidence that caste and cognitive measures explain some of the variation in learning rules, though much of the variation remains unexplained by observable characteristics. Finally, we link individual learning rules with adoption behavior and find that farmers that weight only recent information are less likely to be early adopters.

### 3.1 Data and sampling methodology

**Fig. 1:**
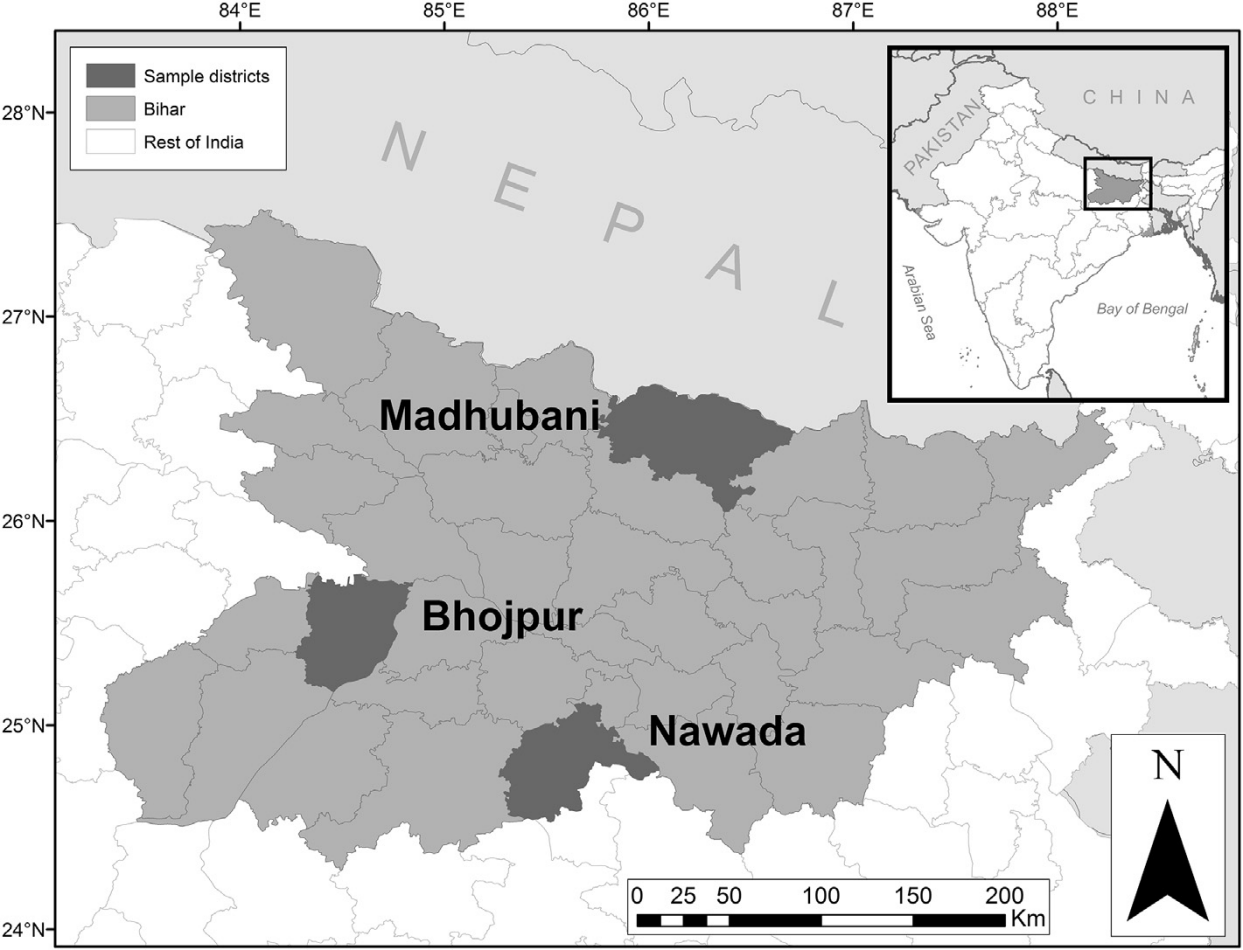
Location of sample districts in Bihar, India.

The data used in this study come from laboratory-in-the-field experiments conducted among rice-producing households in rural Bihar, India during April 2014. The experiments were but one component of a larger, longitudinal data collection effort that gathered, among other things, data on household structure and household member characteristics, seasonal data on land utilization, and season-plot-variety level data on rice production and input use. We employed a multi-stage sampling strategy to generate our sample. In the first stage, we selected three districts heavily depending upon rice production but also displaying significant heterogeneity in terms of agro-ecological conditions. These districts were Bhojpur (west-central Bihar), Nawada (south Bihar, bordering Jharkhand) and Madhubani (north Bihar, bordering Nepal). Fig. 1 illustrates the geographic location of each of these districts. In the second stage, we selected 16 high rice-producing blocks (sub-district administrative units) across the three districts, where the share of blocks drawn from each district was proportional to that district’s share in overall rice production among the three selected districts. Within each of these 16 blocks, we randomly selected two villages. Finally from each of these 36 villages, we randomly selected 18 rice-growing households.

The first round of data collection commenced in April 2013, prior to the kharif (monsoon) season. From this survey, we have detailed information on agricultural production and input use for kharif 2012, along with risk and uncertainty preferences elicited through an experimental approach similar to the one used in Barham, Chavas, Fitz, Rios Salas, and Schechter (2014). In addition, we have information on characteristics of the household head, such as caste, level of education, age, and access to credit. During the follow up survey in April 2014, we gathered information on input usage during kharif 2013 and planned input usage in kharif 2014, which, at the time, was roughly 2 months away. Input and production data is plot specific for up to five of the farmer’s plots on which rice is cultivated. The inputs over which information is collected include irrigation, fertilizers, and seed, and is, in the case of irrigation and fertilizers, collected for each application throughout the season. Plot level characteristics include the slope, soil type, erosion, and contract type. Seed varieties are delineated by name and whether they are local, hybrid, or high yielding varieties. As a result of the two rounds of data collection, we have plot- and time-specific application amounts (planned for kharif 2014 and used during kharif 2013 and 2012).^2^

### 3.2 Experimental procedure

Before describing the experimental procedure for eliciting individual learning rules, we will first briefly introduce the risk and uncertainty experiments that were used to elicit risk and uncertainty preferences, since these preferences play an instrumental role in the identification of learning rules (Ward & Singh, 2015). The identification of risk and uncertainty preferences involved two related experiments conducted as part of the first round of data collection in 2013. In each experiment, farmers were presented with a series of 11 choices between a riskless option and a risky option, which took the form of a simple lottery. The riskless option was always a certain payment of INR 20. The lotteries consisted of a “winning” draw paying INR 40 and a “losing” draw paying some lesser amount. The amount of the “losing” draw monotonically decreases with each subsequent choice. Since this “losing” draw is monotonically decreasing, respondents’ preferences between the risky and riskless options should change at most one time. Following Tanaka, Camerer, and Nguyen (2010), we enforce monotonic preference switching by only asking respondents about the particular choice at which they would switch from preferring the risky option to preferring the riskless option.

Where the risk and uncertainty experiments differ is in the information that is provided to the respondent about the probabilities associated with the “winning” and “losing” draws in the lottery. In the uncertainty experiment, farmers were asked to choose between the risky and riskless options without being provided any information on these probabilities, relying instead on naïve subjective expectations. In the subsequent risk experiment, farmers were told that the odds of “winning” and “losing” the lottery were 50/50. The uncertainty experiment was completed first to ensure that the farmers did not base their beliefs about the distribution of “winning” and “losing” draws based on the probabilities revealed in the risk experiment. To communicate this probability, respondents were shown a bag containing 10 chips, numbered 1 through 10. Chips 1 through 5 were considered to be “winning” draws, while chips 6 through 10 were considered “losing” draws.

Assuming that preferences exhibit constant relative risk and uncertainty aversion (CRRA and CRUA, respectively) with isoelastic utility function $u(c)=\frac{c^{1-\rho }}{1-\rho }$ for ρ≠1 and u(c)=lnc for ρ=1, in which ρ is the CRxA coefficient, the payouts at which the respondent switches from preferring the risky option to the riskless option allows us to estimate an interval of possible risk and uncertainty aversion coefficients. While we can only identify CRxA coefficients within an interval, in the ensuing analysis we make the simplifying assumption that an individual’s risk or uncertainty aversion coefficient takes the value of upper limit of the interval for which the individual’s choices would be consistent.

We attempted to make the choices in these experiments incentive compatible by paying respondents the specified amount for a randomly selected choice situation, depending upon their preference for the risky or riskless option in that particular situation, and, if they preferred the risky option, a random selection of a chip from the bag containing 10 chips.^3^ Table 1 illustrates the series of decisions that participants were asked to make.

**Table 1:** General structure of risk and uncertainty experiments and corresponding coefficients of risk and uncertainty aversion for given switching decision.

		Risky option		Interval for CRxA
		“Winning”	“Losing”	coefficient for switching
Decision	Riskless payout	draw	draw	from risky to riskless
1	INR 20	INR 40	INR 20	(3.76,∞)
2	INR 20	INR 40	INR 16	(1.86,3.76]
3	INR 20	INR 40	INR 13	(1,1.86]
4	INR 20	INR 40	INR 10	(0.65,1]
5	INR 20	INR 40	INR 8	(0.52,0.65]
6	INR 20	INR 40	INR 7	(0.40,0.52]
7	INR 20	INR 40	INR 6	(0.31,0.4]
8	INR 20	INR 40	INR 5	(0.22,0.31]
9	INR 20	INR 40	INR 4	(0.09,0.22]
10	INR 20	INR 40	INR 2	(0.0,0.09]
11	INR 20	INR 40	INR 0	(-∞,0.0]

Note: The interval of CRxA coefficients in the last column was not shown to participants.

The learning experiment was designed in such a way that we are able to identify the most likely heuristic by which individuals form and update beliefs. In this particular experiment, we learn about the rules they employ to update beliefs about the distribution of green and blue beads in a bag containing a total of 100 beads. Before commencing with the actual experiment, farmers were given instructions on the overall structure, goals, and rules of the experiment, as well as being informed of the real financial implications for their decisions. There was also a practice round in which the farmers had the opportunity to practice counting beads and making choices between risky and riskless options (described below). If they chose the risky option during the practice round, the enumerator asked them what their compensation would be if this had been a decision with real financial implications depending upon their draw of a bead from the bag. Even if they chose the risky option, they were subsequently asked to explain what their compensation would have been if they had chosen the riskless option instead and if this decision had real financial implications. The same procedure took place if they initially chose the riskless option. If they made any mistakes in understanding the process or compensation, the enumerator explained the rules again, and with the same beads they had already drawn, asked them to go through the process again. Finally, the enumerator recorded how well the respondents understood the experiment.^4^

The farmers were not aware of the actual distribution of green and blue beads prior to the commencement of the experiment. In each round, the farmers drew five beads out of the bag at random from the bag. After each draw, the number of blue and green beads was recorded on a laminated experiment sheet. The farmers were allowed to contemplate the outcome of the draw before being asked to choose between a riskless option and a risky option. As with the risk and uncertainty experiments, the riskless option consisted of a certain payment of INR 20. The stakes of the risky option were specific to the individual: the high payout (with probability equal to the number of blue beads in the bag) was always INR 40, but the low payout (with probability equal to the number of green beads in the bag) was determined by each farmer’s switching value in the uncertainty experiment described above. Because they do not know the true number of blue and green beads in the bag, their expected payoffs in these situations were determined by their beliefs about the distribution of green and blue beads, based on their current and any previous observations of the draws. After making their choice between the risky and riskless alternatives, the selection was recorded on the same laminated sheet as the number of blue and green beads.

This process continued in the same fashion for 14 rounds. After completion of the 14th round, the farmers were asked to state their belief about the actual number of blue beads in the bag, and were informed that they would be rewarded an extra INR 5 if they were within two of the correct answer (72). After making this guess, the true number of beads was revealed, and farmers were asked one more time to choose between the risky and riskless options. To reduce hypothetical bias, farmers were informed prior to commencing with the experiment that they would be compensated based on one of their choices across the decisions. Their payment was based on a decision that was selected randomly after all decisions were made. If they chose the riskless option in that decision round, they would be INR 20 rupees. If they chose the risky option in that decision round, they were then asked to draw a single bead at random from the bag of blue and green beads to determine their payout from the lottery.

### 3.3 Decision model

Assuming a random utility model, choices reflect utility maximizing behavior with an additive random component. For each choice, agents assess the expected utility difference between choosing the risky option (lottery) and the sure payout using their subjective probabilities to infer the value of the risky option. Assuming that the random component for each choice is independently and identically distributed following an extreme value type 1 (Gumbel) distribution, the choice model in each period reduces to a logit:

(1) $$P(\text{Choose risky option})=\frac{e^{\left(z\right)}}{1+e^{\left(z\right)}},$$

where *z* is the difference in expected utility from choosing the risky option relative to the riskless option.

Consider the case where an individual is faced with a choice over a sure payment of INR 20 and a risky prospect that pays INR 40 if a blue bead is drawn and INR 10 if a green bead is drawn. The expected utility difference between the risky prospect and the sure payment in round *t* is expressed as z(t)=S(t)u(40)+(1-S(t))u(10)-u(20), where S(t) is the individual’s subjective probability of a blue bead in round *t* and u(t) is the utility function, with isoelastic functional form exhibiting CRRA. We estimate the likelihood function using the CRRA value from the risk experiments defined previously.

### 3.4 Learning rules

Individual learning processes are used to inform their subjective value for S(t), where each learning process corresponds to a weighting function that defines the weight of the ith draw of *t* total draws. Then the player’s latent and intrinsic weighting will define the learning rules for each set of choices over *t* draws. While the nature of the experiment and environment are context specific, the literature on learning provides a variety of learning rules that individuals may employ (cf. Gans et al., 2007). To examine different learning processes, we follow the approach used in Barham et al. (2015) and specify four potential models for learning that have legacies in psychological studies of learning patterns. We refer to these four learning processes as Bayesian learning, impressionable learning, reactionary learning, and myopic learning. These learning rules are described in greater detail below.

Consider Bayesian learning, in which information from all rounds is weighted equally and farmers’ subjective probability updates in each round given new information. The Bayesian subjective probability of drawing a blue bead in round $t_{k}$ is therefore

$$S(t_{k})=\frac{1}{t_{k}}\overset{t_{k}}{\underset{t=1}{\sum }}B_{t}\text{.}$$

In contrast, under impressionable learning, farmers only consider the ratio of blue beads from the first *n* rounds when making all of their subsequent choices. We refer to this model of learning as ‘impressionable’ because it reflects the strength of first impressions, in which additional information revealed after the nth round is essentially ignored. In the extreme case, individuals’ beliefs about the distribution are influenced by information revealed in the first – and only the first – round. Under this rule, the impressionable subjective probability in round *t* is given by

$$S(t)=B_{1}\text{.}$$

These are farmers that overweight their initial information and form strong beliefs based on their first impressions, foregoing any future learning.

Similarly, last-*n* learners neglect all of their previous information in favor of only the most recent *n* draws. These farmers may be characterized by having short memories or attention spans, or may simply choose to neglect information beyond the most recent *n* experiences because updating using this relatively distant information may be tedious and error prone. Again, in the extreme, individuals may only attend to the most recent information revealed, ignoring any other information that had previously been revealed. The reactionary learning subjective probability in round *t* is given by

$$S(t)=B_{t}\text{.}$$

Finally, the myopic updating learning rule is similar to last-*n* in that it only considers information from most recent *n* rounds, but uses a simple classification of draws into “good” and “bad.” Draws with 4 or 5 blue beads are considered “good” while draws of 3 or fewer blue beads are “bad.” Farmers characterized by myopic updating learning both overweight only the most recent *n* information and do not fully consider the probabilities over which they are making their choices. The corresponding subjective probability weights are

$$S(t)=\left\{\begin{array}{cc} 1 & “\text{good}”\;\text{draw}\\ 0.5 & “\text{bad}”\;\text{draw} \end{array}\right)$$

Upon calculating these probabilities for each individual, we can fit and evaluate the models based on the Bayes Information Criterion (BIC)=-2∗LL using the calculated logit log-likelihood. We rank each learning rule using the BIC, such that the first best learning rule (i.e., the learning rule that most accurately reflects the observed behavior) has the lowest BIC, the second best learning rule has the second lowest, and so on. Occasionally, learning rules may have equivalent BIC rankings if the sequence of draws and/or decisions are similar. When describing the distribution of these learning rules we include ties between rules in each of their respective totals. After classifying individuals into their first best learning rules, we estimate a multinomial logit model (excluding ties) across the possible first best rules to determine whether literacy, age, caste, and evaluated comprehension of the learning experiment are determinants of particular learning rules. Finally, we model the usage of hybrid rice in 2013 as a function of learning rules and individual characteristics that potentially impact adoption to investigate which learning rules encourage or prevent adoption.

## 4 Results

Table 2 provides household summary statistics for the full sample that participated in the learning experiment and for which we have complete information about their inputs, along with the subsamples of farmers for whom the elicited risk aversion coefficient was finite and infinite.^5^ Column four includes t-tests of the difference between the finite and infinite risk aversion samples. Of the farmers that participated in the survey in 2013, nearly one third (124) had an infinite risk aversion coefficient in the risk experiment. Farmers with a finite risk aversion coefficient had a mean risk aversion coefficient of 0.61, indicating a modest degree of risk aversion. This level of risk aversion is similar to the estimates reported in Binswanger (1981), also from India, though with a slightly different elicitation method. The Binswanger (1981) estimate of 0.71 suggests that the farmers in his sample from semi-arid tracts of Maharashtra and Andhra Pradesh are slightly less risk averse than the farmers in our sample in Bihar. Furthermore, the estimated risk aversion coefficients reported here are roughly consistent with those reported in Cardenas and Carpenter (2008) from many other contexts around the globe. The mean risk aversion coefficient here is slightly lower than those reported by Barham et al. (2015) for Minnesota and Wisconsin farmers (0.77) using a virtually identical preference elicitation mechanism. When generating the likelihoods for risk averse farmers in the following analysis, we exclude those with infinite risk aversion coefficients because we are unable to calculate their utility without using an arbitrary value. While this represents a large portion of the sample, from Table 2 they differ primarily in the caste makeup and evaluated comprehension of the learning experiment.

**Table 2:** Summary statistics for full sample and finite CRRA sample.

		Finite risk	Infinite risk	
	Full sample	aversion sample	aversion sample	Difference
Age	46.90	47.46	45.92	−1.535
	(13.0)	(12.5)	(13.79)	(1.271)
Gender (male = 1)	0.96	0.98	0.94	−0.036^∗^
	(0.20)	(0.15)	(0.24)	(0.018)
Can read and/or write	0.70	0.71	0.66	−0.055
	(0.46)	(0.45)	(0.48)	(0.045)
Comprehension: good	0.44	0.46	0.40	−0.060
	(0.50)	(0.50)	(0.49)	(0.048)
Comprehension: moderate	0.51	0.48	0.58	0.101^∗∗^
	(0.50)	(0.50)	(0.50)	(0.048)
Comprehension: poor	0.04	0.06	0.02	−0.041^∗∗^
	(0.21)	(0.24)	(0.13)	(0.020)
General caste	0.31	0.37	0.21	−0.159^∗∗∗^
	(0.46)	(0.48)	(0.41)	(0.044)
Other backward caste	0.43	0.41	0.46	0.048
	(0.50)	(0.49)	(0.50)	(0.048)
Scheduled caste	0.22	0.20	0.27	0.073^∗^
	(0.42)	(0.40)	(0.44)	(0.04)
Scheduled tribe	0.03	0.02	0.05	0.037^∗∗^
	(0.17)	(0.13)	(0.23)	(0.017)
Access to credit (2013)	0.03	0.03	0.02	−0.016
	(0.17)	(0.18)	(0.13)	(0.016)
Blue ratio (actual)	65.06	65.21	64.80	−0.403
	(11.42)	(11.06)	(12.07)	(1.119)
Blue ratio (guess)	65.35	65.54	65.02	−0.512
	(7.70)	(7.65)	(7.78)	(0.753)
Cultivated hybrid rice (2013)	0.14	0.15	0.13	−0.016
	(0.35)	(0.36)	(0.34)	(0.034)
Risk aversion coefficient		0.61		
		(0.58)		
				
	451	287	164	

Note: ^∗^ Significant at 10% level; ^∗∗^ Significant at 5% level; ^∗∗∗^ Significant at 1% level. Standard deviations in parentheses in columns 1–3, Standard errors in parentheses in column 4.

### 4.1 Rankings of learning rules

We begin by providing rankings of first, second, and third most likely learning rules for the entire sample under the assumption of risk neutrality. These are reported in the panel (a) of Table 3. Due to potential similarities between the weighting functions, we have included ties between rules in both of the corresponding rows. Ties only occurred for first-best rule and were rare, with only one tie in the risk neutral case and three ties in the risk averse case. These results suggest that the most common learning rules are the myopic updating and impressionable learning rules, so that farmers are either reactive to good draws or base much of their following decisions on the first round, implying that first impressions are important for many farmers in the sample. Somewhat surprisingly, Bayesian learning is a more common first ranked learning rule than reactionary learning, and is the most common second-best rule. From this top panel in Table 3, it is clear that there is substantial heterogeneity in the learning rules used by farmers in Bihar, in both their complexity and timing (initial vs. recent information). Despite the low levels of education in the sample, the prevalence of Bayesian learning suggests it may not be a bad approximation for belief updating even in rural villages in developing countries.

**Table 3:** Ranking of learning rules using Bayes Information Criteria.

		(1)	(2)	(3)
		First	Second	Third
(a) Full sample, risk neutral^a^				
	Bayesian learning	18.4	41.7	24.6
	Impressionable learning	33.5	21.1	14.2
	Reactionary learning	14.9	18.8	40.4
	Myopic updating	33.5	18.4	20.8
				
(b) Finite risk aversion sample, risk neutral^b^				
	Bayesian learning	17.8	39.4	24.4
	Impressionable learning	28.9	21.6	17.4
	Reactionary learning	16.0	20.2	37.3
	Myopic updating	37.6	18.8	20.9
				
(c) Finite risk aversion sample, risk averse^b^				
	Bayesian learning	25.4	39.7	25.4
	Impressionable learning	39.0	11.1	9.1
	Reactionary learning	15.3	31.7	33.4
	Myopic updating	21.3	17.4	32.1

Note: For each farmer, the first-best learning rule is the rule with the lowest BIC. The second- and third-best learning rules correspond to their position in the ranking of BIC. In the case of two rules with an equal BIC, both of the learning rules are used to compute the shares provided in the table.

a Sample includes 451 observations.

b Sample includes 287 observations.

The rankings of risk neutral and risk averse learning rules using the limited subsample of farmers with a finite risk aversion are provided in panels (b) and (c) in Table 3, respectively. Surprisingly, the exclusion of farmers with infinite risk aversion does not change the rankings of learning rules under the assumption of risk neutrality relative to those in panel (a). Specifically, we still estimate myopic updating and impressionable learning to be the most prevalent first-best learning rules. However, there are slight differences between the rankings when allowing for risk aversion in the utility functions used in the estimation of the learning rules. As before, fewer farmers exhibit reactionary learning as their first learning rule assuming risk aversion, though this rule may be of secondary or tertiary import, as shown by the increasing frequencies in columns (2) and (3). Notably, while impressionable learning is still prominent (nearly 40 percent of the sample), the number of Bayesian farmers (25 percent) is slightly higher than those relying upon myopic updating (21 percent), though if we consider reactionary and myopic updating learners to be indicative of those with a present bias in their learning, then Bayesian learning is less common than relying upon first impressions or having present biases (36 percent). The fact that myopic updating is more prevalent than reactionary learning, despite the fact that both are biased towards recent observations, suggests that farmers in our sample are more likely to rely upon recent information if it provides a promising signal.

### 4.2 Determinants of learning rules

Next, we investigate whether observable farmer characteristics are correlated with particular learning rules. To do this, we estimate a series of multinomial logits under different sample specifications. Multinomial logit analysis generalizes logit regression to a multiclass setting, essentially allowing the analyst to estimate the marginal contribution of a series of covariates on the probability of a series of outcomes that are generally – though not necessarily – mutually exclusive. We include a set of covariates that may influence the individual’s utilization of a particular learning rule.^6^ Specifically, we include controls for age, gender, literacy, caste, and the enumerators’ evaluations of how well respondents understood the rules and structure of the experiments. The age controls act as a proxy for experience and/or wisdom, which may affect the a farmer’s learning rule through patience and/or years of experience with learning processes. Previous studies have found gender differences in the choice of learning rules which could be the result from innate or social factors that contribute to differences in how men and women either complete the learning experiment or otherwise process information (Barham et al., 2015, Gans et al., 2007). Formal education is relatively low in rural Bihar, particularly among the current generation of adults, though there is variation in the literacy level between farmers. Literacy may contribute to higher-level information processing that carries over into learning about distributions over time. Given the low level of education, enumerator evaluations of respondents’ comprehension provide ancillary controls for intelligence and comprehension that are not accounted for with literacy and how this affects respondents’ strategies in the experiment.

The first set of results in Table 4 are for the full sample under the assumption of risk neutrality. The coefficients for reactionary learning and myopic updating learning suggest that evaluated comprehension is correlated with an individual’s most likely learning rule, as those with poor comprehension are much more likely to use either reactionary or myopic updating learning relative to Bayesian updating, with a relative log odds ratios of 1.7 and 3.6, respectively. Similarly, farmers with only moderate comprehension are more likely to follow myopic updating than Bayesian updating (odds ratio of 1.16), but are less likely to adhere to either impressionable learning (odds ratio 0.71) or reactionary learning (odds ratio 0.50) increase in the relative log odds of using reactionary learning. Taken as a proxy for intelligence, those with lower comprehension exhibit more reactionary responses to favorable draws and are less likely to follow Bayesian updating or use the most recent draws to inform their belief updating. Generally, there is considerable unexplained variation in the choice of learning rules under risk neutrality after controlling for experience, gender and potentially weak proxies for intelligence.

**Table 4:** Multinomial logit relative odds ratios: determinants of first-best learning behavior.

	Full sample, risk neutral			Finite risk aversion sample, risk neutral			Finite risk aversion sample, risk averse		
			Myopic			Myopic			Myopic
Variables	Impressionable	Reactionary	updating	Impressionable	Reactionary	updating	Impressionable	Reactionary	updating
Age	1.003	1.011	1.006	1.015	1.026	1.016	1.020	1.022	1.022
	(0.009)	(0.013)	(0.012)	(0.016)	(0.017)	(0.017)	(0.016)	(0.020)	(0.017)
Male	0.439	0.784	0.527	0.301	0.345	1.693	1.401	1.802	0.479^∗∗∗^
	(0.424)	(0.792)	(0.421)	(0.446)	(0.473)	(2.741)	(1.258)	(2.629)	(0.467)
Can read and/or write	1.255	0.682	1.145	1.802	1.304	2.124^∗^	1.035	0.754	0.763
	(0.359)	(0.242)	(0.366)	(0.740)	(0.625)	(0.864)	(0.428)	(0.390)	(0.352)
Other backward caste	1.016	0.571	0.711	0.860	0.558	0.519	0.469^∗∗^	0.490	0.220^∗∗∗^
	(0.282)	(0.201)	(0.256)	(0.353)	(0.273)	(0.234)	(0.171)	(0.187)	(0.085)
Scheduled caste/tribe	0.948	0.715	0.586	0.771	0.565	0.394^∗^	0.805	0.783	0.701
	(0.442)	(0.371)	(0.260)	(0.426)	(0.356)	(0.210)	(0.336)	(0.426)	(0.404)
Comprehension: moderate	0.708	0.508^∗^	1.060	0.902	0.558	1.196	0.987	0.968	1.638
	(0.179)	(0.198)	(0.284)	(0.261)	(0.276)	(0.397)	(0.335)	(0.464)	(0.584)
Comprehension: poor	0.250	1.782	3.722^∗^	$3.19\times 10^{-7}$^∗∗∗^	1.720	4.375^∗^	0.783	$1.86\times 10^{-7}$^∗∗∗^	0.295
	(0.312)	(1.782)	(2.778)	$\left(2.84\times 10^{-7}\right)$	(1.790)	(3.443)	(0.449)	$(1.26\times 10^{-7}$)	(0.278)
Constant	3.660	1.376	2.752	2.454	1.219	0.465	0.662	0.262	$1.37\times 10^{-7}$^∗∗∗^
	(3.021)	(1.325)	(2.244)	(3.413)	(1.445)	(0.718)	(0.637)	(0.422)	$\left(1.56\times 10^{-7}\right)$
									
Observations	450			286			284		
Log pseudolikelihood	−581.049			−361.099			−361.511		

Note: ^∗^ Significant at 10% level; ^∗∗^ Significant at 5% level; ^∗∗∗^ Significant at 1% level. Standard errors adjusted for clustering at the village level. Bayesian learning is the reference category in all regressions. Caste effects are relative to general caste. Comprehension effects are relative to understanding well. Ties amongst most likely learning rules not included.

The second set of estimates in Table 4 are the multinomial logit estimates under risk neutrality with the subsample of farmers that have a finite risk aversion coefficient. Despite the similarities in the distribution of learning rules and household characteristics, there are evident differences in the determination of learning rules. Surprisingly, the relative log odds of myopic updating learning as opposed to Bayesian learning increase by 2 for farmers that can read and/or write, implying that literacy does not seem to increase the likelihood of more computationally intensive learning rules. Being a member of a scheduled tribe or scheduled caste increases the likelihood of myopic updating learning, but there does not seem to be robust effects of caste across samples under risk neutrality. Farmers with poor comprehension are much more likely to learn according to myopic updating, with relative log odds of 4.18, and slightly more likely to be impressionable learners.^7^ Taken as a current measure of intelligence, farmers with lower evaluated comprehension are more likely to use computationally simple updating rules when making decisions, assuming risk neutrality.

Finally, the third set of estimates in Table 4 are the multinomial logit estimates for the subsample of farmers with finite risk aversion, where we estimate learning rules allowing for individuals to exhibit risk aversion. Across reactionary, impressionable, and myopic updating learning, we find that members of other backward castes (OBC) are more likely to use Bayesian updating (odds ratios of 0.47, 0.49, and 0.22, respectively). Farmers with poor comprehension of the experiment are considerably less likely to be reactionary learners, suggesting that lower comprehension is not necessarily associated with reactive responses to draws.

### 4.3 Accuracy of learning rules

**Fig. 2:**
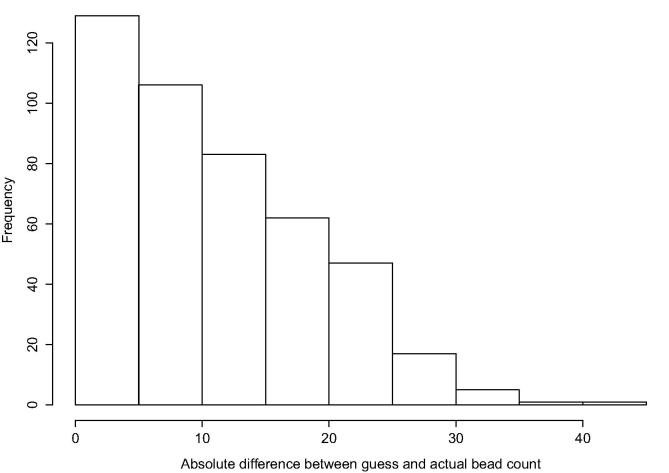
Accuracy of guesses: absolute difference between guess and revealed draws.

After identifying the rankings and determinants of learning rules, we investigate whether particular learning rules are better predictors of individuals’ estimation of the number of blue beads in the bag. Recall that prior to learning the true number of blue beads in the bag, farmers were asked to guess how many they believed were in the bag and were awarded 5 extra rupees if their guess was within two of the actual number. The true number of blue beads in the bag was 72, but farmers observed only a sequence of subsamples over 15 draws (including the practice round). To analyze how accurate farmers were in their estimates, we calculate the absolute difference between their guess and the average of the total draws that they observed. The average is then multiplied by 100 to produce a value on the same scale as the guess. In other words, if an individual observed 57 blue beads during the course of the experiment, then considering there were 75 total beads drawn, the average number of blue beads observed was 0.76. Based on these observations, an individual might reasonably expect that there are 76 blue beads in total in the bag. These absolute differences are illustrated in Fig. 2. We estimate a simple linear regression model with this absolute difference as the dependent variable, conditional on the learning rules and individual characteristics used previously. Village fixed effects are included to control for differences across villages in the time of the survey and potential sharing of information across members in the village of the correct number of blue beads.

Results are provided in Table 5. The first column includes the risk neutral learning rules for the full sample, the second column includes the risk neutral learning rules for the subsample with finite risk aversion, and the third column includes the risk averse learning rules for the subsample with finite risk aversion. Bayesian learning is the excluded rule in all three columns. Myopic updating learners perform significantly worse in their guesses than Bayesian learners regardless of the subsample or risk aversion, with the average guess nearly 3 beads further away from the actual number of blue beads compared with Bayesian learners’. Given that myopic updating learners base their beliefs on promising signals (i.e., only rely on information if there were 4 or 5 blue beads drawn), this likely leads to overestimation of the number of blue beads, since their mean posterior belief is on the order of 80 blue beads or more. In contrast, reactionary learners perform significantly better than Bayesian learners. Reactionary learners’ guesses, on average, are nearly 4 beads closer to the actual amount than Bayesian learners. We note that there is little evidence to suggest that, after controlling for individual learning rules, that perceived comprehension affects performance in guessing the actual number of blue beads. Across all three regressions, those individuals who did not appear to fully comprehend the game perform no worse than those who do.

**Table 5:** Difference in guess from revealed probability in learning game.

		Finite risk	Finite risk
	Full sample,	aversion sample,	aversion sample,
	risk neutral	risk neutral	risk averse
Impressionable	−0.690	−0.479	−0.454
	(1.138)	(1.460)	(1.463)
Reactionary	−3.804^∗∗∗^	−4.079^∗∗∗^	−4.075^∗∗∗^
	(1.310)	(1.560)	(1.562)
Myopic updating	3.000^∗∗∗^	3.052^∗∗^	3.094^∗∗^
	(1.139)	(1.431)	(1.441)
Comprehension: moderate	1.115	1.301	1.339
	(0.767)	(1.004)	(1.010)
Comprehension: poor	−0.613	0.964	0.983
	(1.840)	(2.156)	(2.158)
			
Observations	450	286	284
Pseudo-R^2^	0.152	0.222	0.221

Note: ^∗^ Significant at 10% level; ^∗∗^ Significant at 5% level; ^∗∗∗^ Significant at 1% level. Robust standard errors in parentheses. Bayesian learning is the reference category in all regressions. Comprehension effects are relative to understanding well. Ties amongst most likely learning rules not included. All regressions contain intercepts, controls for caste, access to credit, age, gender, and literacy, as well as village fixed effects.

### 4.4 Learning and technology adoption

Finally, we investigate the relationship between learning rules and the decision to adopt a new agricultural technology, specifically hybrid rice. In many ways, rice hybrids represent the next generation of the Green Revolution. Particularly in light of the world food price crisis in 2007–08, there has been increasing interest in many developing countries for finding solutions to increase productivity growth to ensure food security, and there are hopes that hybrid rice might be one such solution (Spielman et al., 2013, Spielman et al., forthcoming). Previous research suggests that hybrids have had a significant effect on improving livelihoods and food security in several developing countries – most notably China – where rice is the principal foodgrain (e.g. Janaiah et al., 2002, Lin and Pingali, 1994). Hybrids can contribute to increased food security both for producers (through higher productivity, resulting in increased own-consumption as well as larger marketable surpluses, which in turn results in higher farm incomes) as well as consumers (the increased quantity of rice on the market results in lower and more stable prices). Hybrids typically have considerably higher yields than conventional inbred varieties – even later generations of modern, high-yielding varieties arising from the Green Revolution. Much of these higher yields can be attributed to heterosis – the increase in the vigor of the rice crop resulting from the genetic contributions that result from crossing distinct parental lines. Not only does heterosis typically confer higher yields, but there is also a significant increase in genetic uniformity, which translates into an economic benefit through a lower seed rate needed to cultivate a given area (typically about 1/3).

Despite these benefits, rice hybrids are not without significant downsides. Perhaps the most glaring detraction is the significantly higher seed price (on the order of about 10 times the price of modern varieties). This higher seed price is partially offset by the lower seeding rate, but these higher seed prices – in conjunction with typically increased expenditures on complementary inputs like fertilizer and irrigation – still often results in higher operational costs for hybrid rice production compared to cultivating modern varieties. Furthermore, yields and genetic uniformity decline dramatically after the first generation of seed (F1). There is therefore no benefit to farmers to save and store harvested grains to use as seed in subsequent seasons. Rather, farmers must typically purchase new F1 seed on a continual basis if they wish to avail the benefits of hybrids.

An analysis on the economics of hybrid and conventional rice production in Bangladesh – which has significantly more experience with hybrids than India – demonstrates many of these factors, both pro and con (Azad, Mustafi, & Hossain, 2008). Over two years, this study demonstrates a consistent 24 percent higher grain yield for rice hybrids compared to conventional varieties. Hybrid rice seeds were between 65 and 190 percent higher than for conventional varieties, but the quantity of seed required was between 77 and 83 percent less for hybrids. While the total costs associated with cultivating hybrids were higher than for conventional varieties, the net returns were between 30 and 50 percent higher for farmers cultivating hybrids.

The government of India has set ambitious targets to increase the area under hybrid rice. As part of its National Food Security Mission, the goal was to increase the area under hybrid rice to as much as 25 percent of all cultivated rice area by 2015, up from only about 6 percent in 2008–09 (Spielman, Kolady, Ward, Ar-Rashid, & Gulati, 2012). As with any relatively new technology, however, farmers’ decision to ultimately adopt hybrids depends crucially on subjective beliefs about the profitability of cultivation, and how farmers formulate and update these beliefs with exposure to new evidence. From the summary statistics reported in Table 2, we see that some farmers in our sample have adopted rice hybrids, but the expansion is far from complete, with only 15 percent of farmers having adopted. We do not have a complete history of hybrid crop usage, so unfortunately we are unable to focus on the timing of the decision. Thus, our estimation of the effects of different learning rules on hybrid adoption includes only a snapshot of “earlier adopters” and potential determinants of adoption.

We estimated the decision to use hybrid rice on any of the farmers’ rice plots during kharif 2013 using a simple probit model conditional on the different learning rules and other covariates. Access to credit is included as a covariate in this model because access to credit can alleviate cash constraints and allow farmers to purchase the more expensive hybrid seeds.^8^ Marginal effects from estimating this probit model using maximum likelihood are reported in Table 6.^9^ As before, columns 1 and 2 include the estimates in which learning rules are estimated under the assumption of risk neutrality using the full sample and the subsample of farmers with finite risk aversion, respectively. Column 3 includes the learning rules estimated with risk aversion. All three models explain between roughly 35 and 40 percent of the variation in hybrid adoption, with the best fit arising in Column 3 when risk aversion is accounted for. Across all three regressions, village fixed effects are included to control for unobservable heterogeneity at the village level that may affect the decision to use hybrid rice during kharif 2013. The sample size decreases by nearly half using the village fixed effects due to many of the villages having zero adoption.

**Table 6:** Probability of cultivating hybrid rice in kharif 2013 (marginal effects from probit regression).

		Finite risk	Finite risk
	Full sample,	aversion sample,	aversion sample,
	risk neutral	risk neutral	risk averse
Impressionable	−0.089	−0.140^∗^	−0.269^***^
	(0.057)	(0.082)	(0.057)
Reactionary	−0.212^***^	−0.287^***^	−0.293^***^
	(0.045)	(0.049)	(0.047)
Myopic updating	−0.010	−0.041	−0.107
	(0.062)	(0.085)	(0.081)
Credit	0.165	0.344^**^	0.451^***^
	(0.157)	(0.173)	(0.098)
Comprehension: moderate	−0.087^∗^	−0.036	−0.078
	(0.046)	(0.072)	(0.069)
Comprehension: poor	−0.095	−0.046	−0.201^***^
	(0.093)	(0.145)	(0.069)
			
Observations	233	136	136
Pseudo-R^2^	0.374	0.341	0.386

Note: ^∗^ Significant at 10% level; ^∗∗^ Significant at 5% level; ^∗∗∗^ Significant at 1% level. Standard errors computed using the delta method reported in parentheses. Bayesian learning is the reference category in all regressions. Comprehension effects are relative to understanding well. Ties amongst most likely learning rules not included. All regressions contain intercepts, controls for caste, age, gender, and literacy, as well as village fixed effects.

Across all three columns in Table 6, we observe that farmers that are characterized by reactionary learning in the learning experiment are less likely to be early adopters relative to Bayesian learners. Thus, farmers that have more present biased learning processes (reactionary learning) are less likely to be early adopters even after controlling for farmer comprehension, which was previously shown to be negatively correlated with reactionary learning. When learning rules are estimated assuming risk aversion (column three), we find that impressionable learners are similarly less likely to adopt hybrid rice compared with Bayesian learners. These farmers rely upon first impressions, so may be heavily influenced by the high up-front cost of hybrid seeds. The fact that Bayesian learners are generally more likely to adopt hybrids than those that rely upon other learning rules is not terribly surprising. With new technologies, including new seeds, there is typically a process of tinkering and making marginal adjustments to learn about the appropriate use of both the technology itself as well as complementary inputs. Bayesian learners are much more suited for this type of process than those that are either present biased or those that rely upon first impressions. But Bayesian learning is also considerably more cognitively taxing, as it requires a much longer memory and a more complex updating process. It is not surprising, therefore, that most farmers within our sample rely on less complicated learning rules. But the fact that over 25 percent of the farmers in our sample rely upon Bayesian learning as a first-best description of their learning process suggests that models assuming such learning may provide reasonable predictions and testable hypotheses about farmer behavior, even in rural settings in developing countries like India.

Unsurprisingly, credit access is positively correlated with early adoption of hybrid rice for the subsample of farmers with finite risk aversion coefficients, despite the overall level of credit access being incredibly low (only about 3 percent of farmers in our sample reported accessing credit during kharif 2013.^10^ Farmers with lower evaluated comprehension in the experiment – which may be a reasonable proxy for intelligence – are less likely to be early adopters of rice hybrids, implying that a measure of current intelligence, as opposed to past experience in education, may be an impediment to adoption independent of learning processes.

## 5 Conclusion and discussion

In this paper, we have used experimental methods to identify various processes by which farmers in rural Bihar formulate and process information. Our results suggest that there is a great deal of heterogeneity in farmers’ learning heuristics, both in complexity and in the way they rely upon initial versus recent information. Generally, our results suggest that farmers in our sample tend to either rely upon first impressions or react to recent information. Farmers are more likely to react to recent information if it provides a promising signal as opposed to a simple, ambiguous message. Despite this tendency, however, we also find that roughly a quarter of the farmers in our sample can best be described as Bayesian learners, in which each past observation is used to inform posterior beliefs. This is an interesting finding, given the cognitive tax that such processing imposes. Given that researchers often assume Bayesian learning processes in models of technology adoption, these results suggest that such models may provide reasonable predictions about farmer behavior.

The heterogeneity in learning patterns that we observe is difficult to ascribe to observable individual characteristics. In some ways, this suggests that learning rules are intrinsic, and are not systematically determined by gender or age or, with some exceptions, by caste. However, we find convincing evidence that the nature of learning does impact technology adoption, with Bayesian learners typically much more likely to adopt hybrid rice than those characterized by impressionable or reactionary learning. Unfortunately, the lack of observable characteristics associated with learning styles makes targeting based on learning styles difficult.

What do our results imply for Indian agricultural policies, particularly around hybrid rice, which the Indian government views as an important technology in its plans to increase food security? Our results suggest a continued need for formal financial integration in rural India, since access to credit significantly increases adoption of hybrid rice, and likely has similar effects with other agricultural technologies. Furthermore, given the heterogeneity in learning rules that we observe in our sample, efforts to promote hybrids (or likely any new technology) will likely require a more nuanced approach, rather than a one-size-fits-all extension message. Given the high share of farmers in our sample that rely on first impressions when learning, any approach to introduce hybrid seeds, or new technologies, should take into account the impacts that poor first impressions have for further adoption. Extension agents or private firms should plan demonstration plots or sample seed pack distributions in seasons in which the technology will have positive impacts to the best of their ability. In addition, extension agencies should focus on providing sufficient resources during initial seasons to ensure that the technology performs as well as possible. These resources potentially include information on optimal cultivation practices for the technology, provision of inputs, and proper management of demonstration plots. While the subset of Bayesian or present biased farmers would be more likely to forgive earlier failures if they are followed up by promising signals, the longer term impacts for impressionable farmers justify higher initial costs and properly timed introduction of technologies. It is yet to be seen whether access to credit augments in any way the effects of the various learning patterns, but if so, there may be further implications for the pathways by which information is conveyed, perhaps by imposing conditions on access to credit that may increase the likelihood of sustained adoption.

Due to the idiosyncratic nature of learning that we observe, it would be difficult to prescribe individualized messages or interventions to farmers based solely on observable characteristics. However, depending on the nature of the technology, pilot surveys that measure the share of learning styles by village may be a worthwhile investment for planning future interventions. Further, understanding learning behavior and trying to determine observable characteristics that correlate with learning style would be a fruitful area for future research so that extension agents can make better decisions about when and where to roll out new technologies. Given cost constraints, targeting villages for rollout of technologies based on their share of impressionable farmers may allow extension agents to spread out the risk of a poorly performing season. Efforts to collect information on learning styles at the village level over a larger area will also contribute to our understanding of individual or environmental characteristics that lead to heterogeneity in learning. Further, identifying appropriate messaging strategies to satisfy the needs of different learners may be a fruitful avenue of future research.

## Footnotes

1 Traditionally, it was thought that such decisions were made on the basis of net present value. Carey and Zilberman (2002), for example, have suggested that one source of heterogeneity in technology adoption may arise due to differences in the option value ascribed to postponing investment in the new technology (i.e., differences in the critical value above which the expected long-term value of adoption must exceed the initial investment).

2 For households that cultivated hybrids, we do not have information on the timing of adoption, only whether they cultivated during the previous two kharif seasons. This limits our ability to say anything about learning effects over time or potential disadoption, but given the relative novelty of hybrids in India, we do not feel this is a serious deficiency.

3 In reality, there were three other similar experiments that were conducted during the same interview, so the potential payout was not limited to these two experiments.

4 Clearly, the practice round provided some information about the number of beads in the bag, and therefore the learning rules are calculated including this information as the first round in the updating of subjective probabilities.

5 As previously stated, the risk aversion coefficients elicited through the experimental procedure documented above fell within an interval. To operationalize analysis, we assumed that an individual’s coefficient of risk aversion was the value at the upper limit of this interval. This implied that some individuals (i.e., those individuals who preferred the riskless option in first round of Table 1) were assigned an infinite coefficient of risk aversion. Given that the expected value of the lottery is INR 30 and, even with a “losing” draw, the lottery payout would be no less than the riskless option, individuals choosing the riskless option clearly display an considerable aversion to risk. Whether it is fair to characterize these individuals as infinitely risk averse is debatable. Yet it is also not straightforward to study their information processing strategies under such extreme preferences. We do not believe that this demonstrates a deficiency in our experimental design. Rabin (2000) and Rabin and Thaler (2001) have noted that moderate risk aversion in medium stakes gambles should imply high risk aversion in higher stakes gambles. Given the moderate stakes in our experiment, the high risk aversion among these farmers must surely be indicative of an absolute disdain for risk, which is perhaps appropriately reflected in an infinite coefficient of relative risk aversion.

6 We exclude the one farmer with a tie in their first best rule so the rules are mutually exclusive.

7 While the relative odds ratio is statistically significant with very low probability of Type I error, the effect size is so small as to likely not be a particularly relevant determinant of learning behavior.

8 Access to credit was quite low in our sample, which would suggest the incompleteness of markets and thus the non-separability of production and consumption decisions at the household level. We abstract from the issue of separability and maintain an assumption of separability to illustrate the role of learning heuristics on production decisions, though we acknowledge this is a limitation of the present study.

9 The estimated coefficients from these probit regressions are reported in Table A in the Appendix.

10 As a result of this low level of credit access, we are unable to ascertain whether credit access introduces heterogeneity in the effects of the various learning rules discussed in this study.
