# Supplementary material for: Can differences in individual learning explain patterns of technology adoption? Evidence on heterogeneous learning patterns and hybrid rice adoption in Bihar, India
Source: World Dev. 2019 Mar;115:178–89. doi: 10.1016/j.worlddev.2018.11.014 (PMC6333296; doi:10.1016/j.worlddev.2018.11.014)
Supplement: Supplementary file 1 [file mmc1.pdf]

A Supplemental tables

Table A1: Probability of cultivating hybrid rice in kharif 2013

	Full sample, risk neutral		Finite risk aversion sample, risk neutral		Finite risk aversion sample, risk neutral	
	Estimate	Std. Error	Estimate	Std. Error	Estimate	Std. Error
Impressionable	−0.440	0.286	−0.675	0.430	−1.551	0.417
Reactionary	−1.442	0.432	−2.079	0.601	−1.982	0.482
Myopic updating	−0.048	0.302	−0.189	0.400	−0.584	0.493
General caste	−0.273	0.262	−0.453	0.339	−0.787	0.370
Other backward caste	−2.089	0.617	−3.712	0.991	−3.364	0.859
Credit	0.706	0.619	1.425	0.774	2.110	0.621
Age	0.012	0.009	0.021	0.013	0.024	0.012
Male (=1)	0.537	0.697	0.919	0.877	1.139	0.905
Can read/write	−0.007	0.279	−0.279	0.420	−1.081	0.432
Comprehension: moderate	−0.423	0.224	−0.166	0.326	−0.385	0.341
Comprehension: poor	−0.527	0.602	−0.218	0.723	−1.308	0.641
Village fixed effects	Yes		Yes		Yes	
Observations	Yes		Yes		Yes	
Pseudo-R^2	Yes		Yes		Yes	

Note: * Significant at 10% level; ** Significant at 5% level; *** Significant at 1% level. Robust standard errors in parentheses. Bayesian learning is the reference category in all regressions. Comprehension effects are relative to understanding well. Ties amongst most likely learning rules not included. All regressions contain intercepts, controls for caste, age, gender, and literacy, as well as village fixed effects.
